# Exercise-induced left septal fascicular block: an expression of severe myocardial ischemia

**Published:** 2006-04-01

**Authors:** Augusto Hiroshi Uchida, Paulo Jorge Moffa, Andrés Ricardo Pérez Riera, Beatriz Moreira Ayub Ferreira

**Affiliations:** * Heart Institute of the University of Sao Paulo, Sao Paulo - Brazil; † ABC Medicine Faculty - ABC Foundation - Santo André - Sao Paulo - Brazil

The electrocardiogram (ECG) criteria for the left septal fascicular block (LSFB) are not universally accepted and many other denominations can be seen in literature: focal septal block, septal focal block, left septal fascicular block, left anterior septal block, septal fascicular conduction disorder of the left branch, left septal Purkinje network block, left septal subdivision block of the left bundle branch, anterior conduction delay, left median hemiblock, left medial subdivision block of the left bundle branch, middle fascicle block, block of the anteromedial division of the left bundle branch of His, and anteromedial divisional block. During exercise stress test, fascicular blocks (left anterior and posterior) seem to indicate severe coronary artery narrowing of left main coronary or proximal left anterior descending artery disease [1] and transient exercise-induced left septal fascicular block has been reported a few times [2,3].

54-year-old male, with a history of essential arterial systemic hypertension, primary hyperlipidemia and six-month typical chest pain during exercise (Class II - Canadian Cardiovascular Society) underwent an exercise stress test. During the exercise stress test, ECG demonstrated abrupt prominent anterior forces, an increase in R wave amplitude from V1 to V4, extreme left axis deviation and minor ST segment depression in DII, DIII and aVF (Figure 1). The post-exercise period showed progressive return of the QRS axis in both frontal and horizontal planes and the ST depression worsened by 1 mm. Coronary angiogram (Figure 2A) showed a critical proximal left anterior descending artery lesion. An exercise stress test done three months after coronary artery bypass surgery grafting was normal (Figure 2B).

## Discussion

Intraventricular conduction abnormalities consistent with LSFB are exceptionally recognized. Its diagnosis requires particular attention to changes in the direction of the initial and middle forces of ventricular activation in right and/or middle precordial leads. We divided ventricular activation of LSFB into four successive moments:

1. Initial vector of 20 ms (named 1PI vector) as a consequence of left anterior fascicular block (LAFB) and LSFB. The onset of ventricular activation will depend on the division that is not blocked; i.e. the left posterior-inferior fascicle that ends in the base of the postero-medial papillary muscle of the mitral valve in the postero-inferior region of the left ventricle. The activation of this area originates the so-called 1PI vector (the initial 20 ms vector) directed to left and often to the back, frequently manifesting initial embryonic q waves from V1 to V3.
2. Vector from 20 ms to 40 ms: it represents the complete activation from right to left of the postero-inferior region of the left ventricle; originating the so-called 2PI vector, heading backward, downward and to the left;
3. Vectors from 40 ms to 60 ms: from 40 ms the blocked antero-superior and antero-medial regions activate. This activation is processed through septal and free-wall Purkinje arborizations, which interconnect the three areas. This phase is represented by us with the modified names as 2AS and 2AM vectors. The first one causes the classical pattern of left anterior fascicular block in the frontal plane and the second one prominent anterior forces in the horizontal plane, translated by R waves of increased voltage "in crescendo" from V1 to V3 and decreasing from V5 to V6.
4. Basal vector beyond 60 ms: it corresponds to activation of the basal region of both ventricles, being called basal vector.

**Figure 1:**
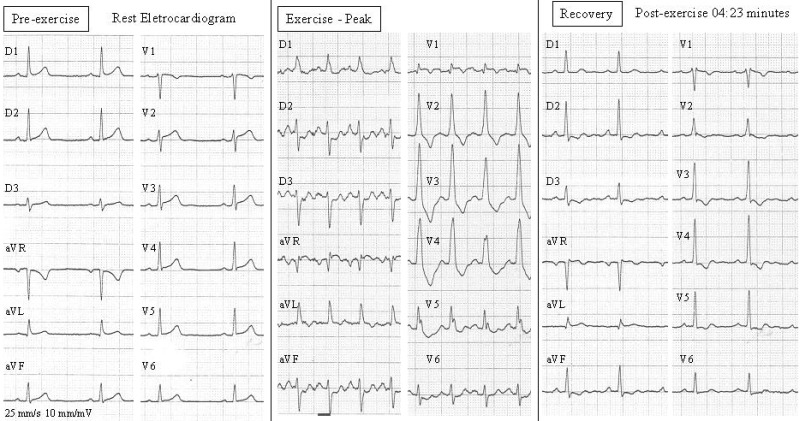
Tall R waves in V1-V4 can be a normal variant in only 1% of patients1 and it is a hallmark ECG finding in left septal fascicular block. The proposed ECG criteria for LSFB are: prominent R waves in V1-V3, minimal QRS prolongation (QRS < 120 ms), T wave morphologic alteration (flat or inversion: very debatable and variable), frequent initial q wave in right and/or middle precordial leads and clinical absence of other causes of prominent anterior forces.

The peak exercise tracing (Figure 1) bears similarity to "standard masquerading" bundle branch block, in which the limb leads look like left bundle branch block (LBBB) and precordial leads like right bundle branch block (RBBB). The QRS duration increased from about 80 ms to 120 ms, and time to peak of R wave in V1 from 20 ms to 70 ms. But, if we observe in detail the left limb leads, DI and aVL, there is no final S wave in them (the QRS complex is a pure R), which leads us to think that these prominent anterior forces are not due to a lesser degree of RBBB, but to LSFB. An isolated RBBB (without LAFB) always present S waves in the left sided leads. On the other hand, in this case there are no final S wave in the left leads (Figure 1 recovery - post-exercise 04:23 minutes).

Differential diagnosis of prominent anterior forces (tall R waves in right and/or middle precordial leads) must include3-6: normal variant, misplaced precordial leads, right bundle branch block, left ventricular ectopy, right ventricular enlargement, Wolff-Parkinson-White with posterior accessory pathway, strictly posterior and posterolateral myocardial infarction, obstructive and non-obstructive forms of hypertrophic cardiomyopathy, progressive muscular dystrophy, dextrocardia, and associations of the previous ones. In this case, the basal ECG did not show a tall R wave from V1 to V4. The exercise-induced transient prominent anterior forces that we interpreted as being secondary to LSFB showed as an abrupt transient tall R wave from V1 to V4, without concomitant significant ST depression. After myocardial revascularization this phenomenon was not found on exercise stress test reflecting the ischaemic nature of this abnormality manifested mainly as dromotropic disorders.

## Figures and Tables

**Figure 2A:**
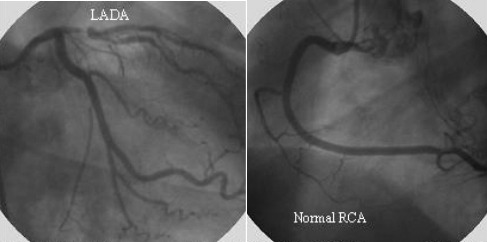
Critical lesion in proximal portion of the left anterior descending coronary artery (LADA)

**Figure 2B:**
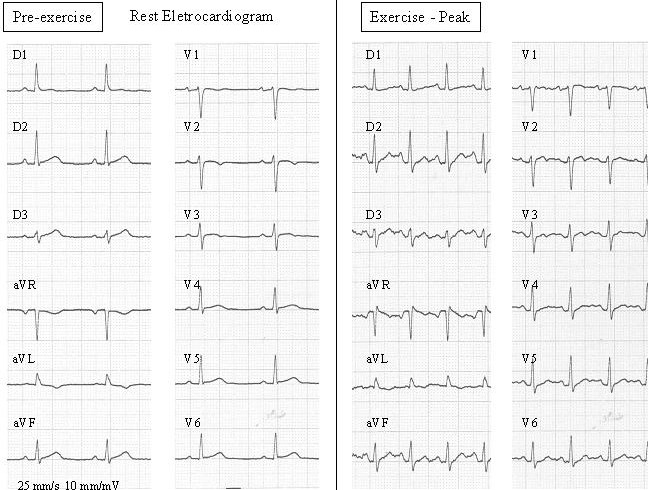
Normal exercise testing, after sucessful coronary bypass graft revascularization
